# Symptomatic Coronary Anomalies and Ischemia in Teenagers – Rare but Real

**DOI:** 10.3389/fcvm.2020.559794

**Published:** 2020-09-22

**Authors:** Julia Borns, Christoph Gräni, Alexander Kadner, Martin Gloeckler, Jean-Pierre Pfammatter

**Affiliations:** ^1^Department of Cardiology, Center for Congenital Heart Disease, Inselspital, Bern University Hospital, University of Bern, Bern, Switzerland; ^2^Department of Cardiology, Inselspital, Bern University Hospital, University of Bern, Bern, Switzerland; ^3^Department of Cardiovascular Surgery, Centre for Congenital Heart Disease, Inselspital, Bern University Hospital, University of Bern, Bern, Switzerland

**Keywords:** coronary artery anomaly, anomalous aortic origin of the coronary arteries, AAOCA, chest pain, interarterial, angina pectoris, teenager

## Abstract

Three cases of teenagers with anomalous aortic origin of the coronary arteries (AAOCA) are presented with typical exercise induced symptoms (chest pain, syncope or dizziness). Using multimodal imaging, diagnoses was confirmed showing interarterial and/or intramural course of the coronary artery explaining the ischemia induced symptoms. Successful surgical correction with unroofing of the AAOCA was performed in all three cases with a favorable outcome. Even though AAOCA are rare, some variants may be relevant and potentially life threatening, therefore treating physicians should be aware of correctly diagnosing and treating these individuals.

## Introduction

Anomalous aortic origin of the coronary arteries (AAOCA) is a rare but potentially life threatening congenital heart disease, especially in the young. Most frequently in this anomaly, the left coronary artery arises from the right-facing sinus or the right coronary artery arises from the left-facing sinus or either coronary artery originating from the non-coronary sinus. Depending on the subsequent course of the anomalous coronary artery, symptoms and relevance can vary. The danger being a fixed narrowing or a dynamic lateral compression of the coronary artery with subsequent myocardial ischemia. Most variants are benign anomalies with only rare reported cases of ischemia. Nevertheless, the subtype with interarterial and/or intramural course of coronary artery is associated with myocardial ischemia and sudden cardiac death (1). Typical symptoms are exercise induced chest pain, dizziness or syncope, but in about half of patients with AAOCA its first presentation is sudden cardiac arrest (2). Using multimodality cardiac imaging aims to characterize the exact anatomy of the anomalous coronary artery and is a central part in the interdisciplinary decision-making toward surgical correction (3). Therefore, it is crucial to identify patients at risk, in order to provide optimal care prior to irreversible myocardial damage. Here, we present three cases of symptomatic teenagers with AAOCA, and explore the challenges we are facing when teenagers present with symptoms suggestive of AAOCA. Further, we show how multimodal imaging helped in diagnosing and the decision-making toward surgery and explore on the details of the surgical approach of unroofing.

## Case Series

### Case 1

A 14-year-old teenager was admitted to our emergency department with exercise induced chest pain. While at the local swimming pool with his school and running multiple times a flight of stairs to the upper level, he developed chest pain, nausea and dizziness. When the ambulance arrived, he was in a hemodynamically stable condition and the pain slowly subsided. In the medical history, he reported two similar episodes within the last 6 month with acute chest pain and dizziness after strenuous exercise. Because of rapid improvement of symptoms after ceasing exercise, no medical care was sought following these earlier episodes. In rest conditions and under moderate physical exercise he was completely asymptomatic and he described himself as having a normal level of fitness. The further past medical history and family history was unremarkable.

**Figure 1:**
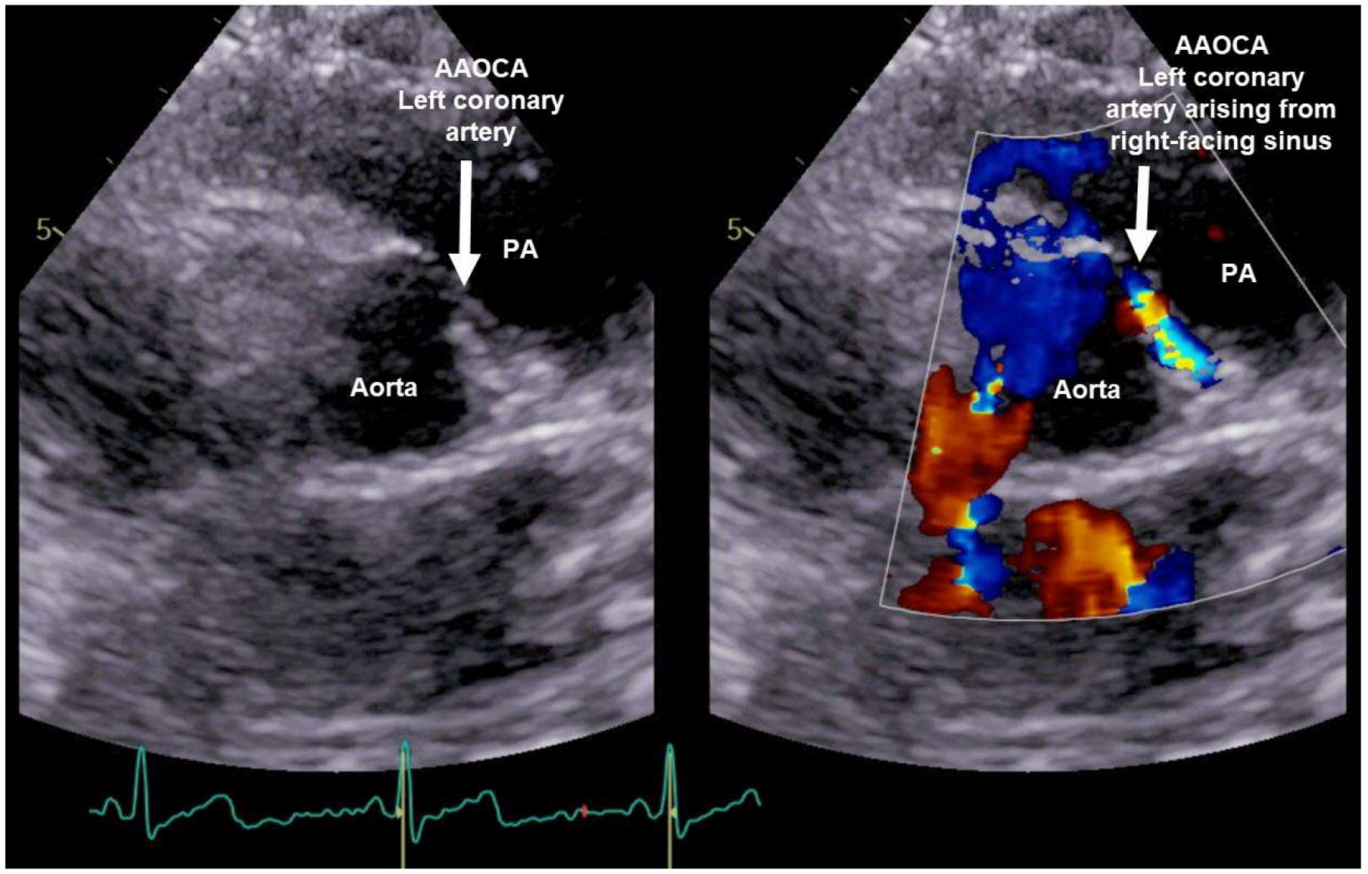
Echocardiography of patient 1: Coronary flow pattern arising from the right-facing sinus and continuing between aorta and pulmonary artery (PA) suggesting an interarterial/intramural course of the left coronary artery.

At time of admission to the emergency department, the vital parameters were within the normal range. The ECG did not show any abnormalities. The laboratory tests showed an elevated high sensitive Troponin T (TroponinThs) of 309 ng/L (reference value <14 ng/L) and slightly elevated CK-MB of 5.9 μg/L (reference value <4.9 μg/L). In the echocardiography, a coronary artery anomaly was suspected, because the left coronary artery did not arise from the left-facing sinus and the presence of a coronary flow pattern arising from the right-facing sinus and continuing between aorta and pulmonary artery suggesting an interarterial course of the left coronary artery. Left- and right heart dimensions and functions were normal (Figure 1).

**Figure 2:**
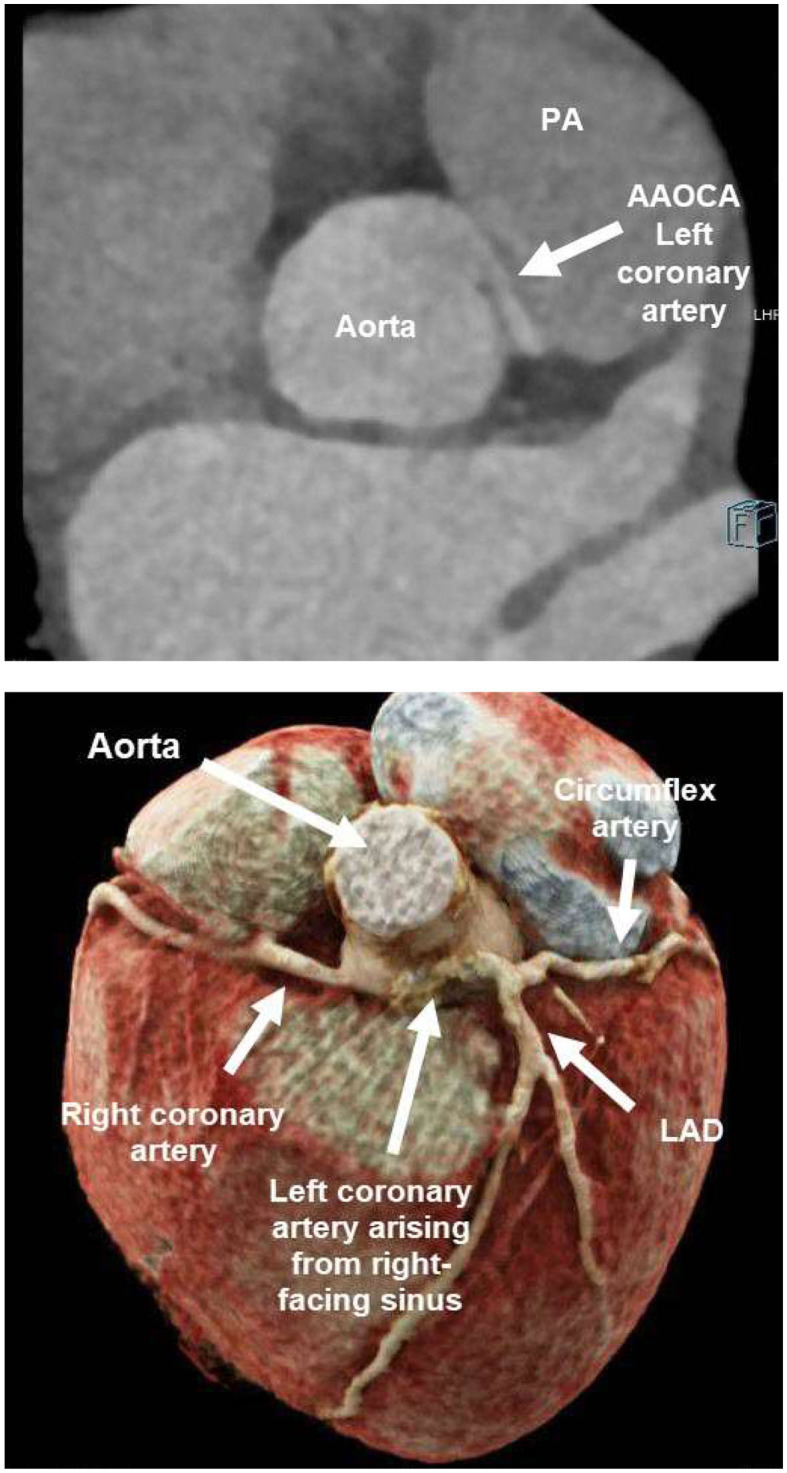
Ultra-low dose coronary computed tomography angiography (CCTA) of patient 1: anomalous aortic origin of the left coronary artery (LCA). LCA arising from the right-facing sinus with acute take-off angle, slit-like ostium and interarterial/intramural course between aorta and pulmonary artery (PA). Normal branching in left anterior descending (LAD) and circumflex artery.

An ultra-low dose coronary computed tomography angiography (CCTA) with 0.38 mSv and 60 ml contrast agent was performed and proved the suspicion of an anomalous aortic origin of the coronary artery (AAOCA) with the left coronary artery arising from the right-facing sinus with an acute take-off angle, a slit-like ostium and an interarterial and intramural course between the aorta and pulmonary artery (Figure 2). The further course of the left coronary artery with branching in left anterior descending (LAD) and circumflex artery was normal, as well as the origin and course of the right coronary artery (RCA).

**Figure 3:**
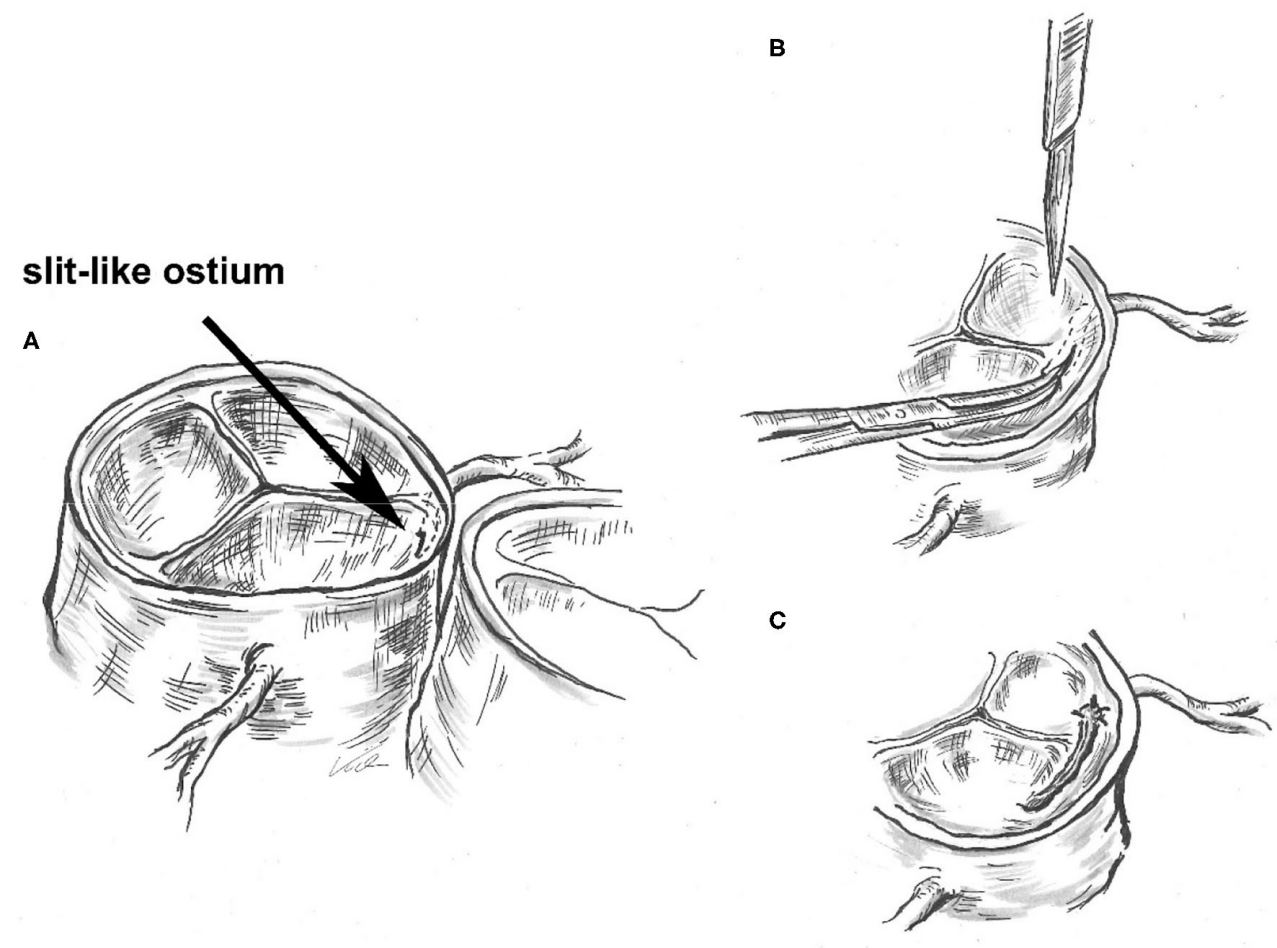
Surgical unroofing of the left coronary artery (LCA): Identification of the slit-like ostium of the LCA in the right- facing sinus and its intramural course toward the left-facing sinus **(A)**. Unroofing of the LCA with a longitudinal incision into the intima from its ostium up to its off-spring from the aortic root **(B)**. By this, enlarging the ostium and eliminating the dynamic obstruction of the proximal main stem of the LCA **(C)**.

Because of this symptomatic AAOCA with ischemia, there was an indication for a timely operation. Preoperatively, the patient showed no further symptoms, and Troponin T and CK-MB levels declined in the serial laboratory testings. Five days after diagnosis, the patient underwent surgical correction (Figure 3). Following installation of the heart-lung machine and cardiologic arrest of the heart, the aorta was opened at the level of the sinotubular junction. The slit-like ostium of the LCA in the right- facing sinus and its intramural course toward the left-facing sinus was identified. Over an inserted coronary probe, the LCA was unroofed with a longitudinal incision into the intima from its ostium up to its off-spring from the aortic root. By this, the ostium was enlarged and the dynamic obstruction of the proximal main stem of the LCA was eliminated. Operation and postoperative course were without complication and the patient recovered quickly. ECG and echocardiography did not show any signs of ischemia and the patient was discharged after 6 days.

In regular follow-ups, the patient is recovering well and has not reported any symptoms of ischemia, at rest or while strenuously exercising. ECG and echocardiography findings remained unremarkable during follow-up on day 30.

### Case 2

**Figure 4:**
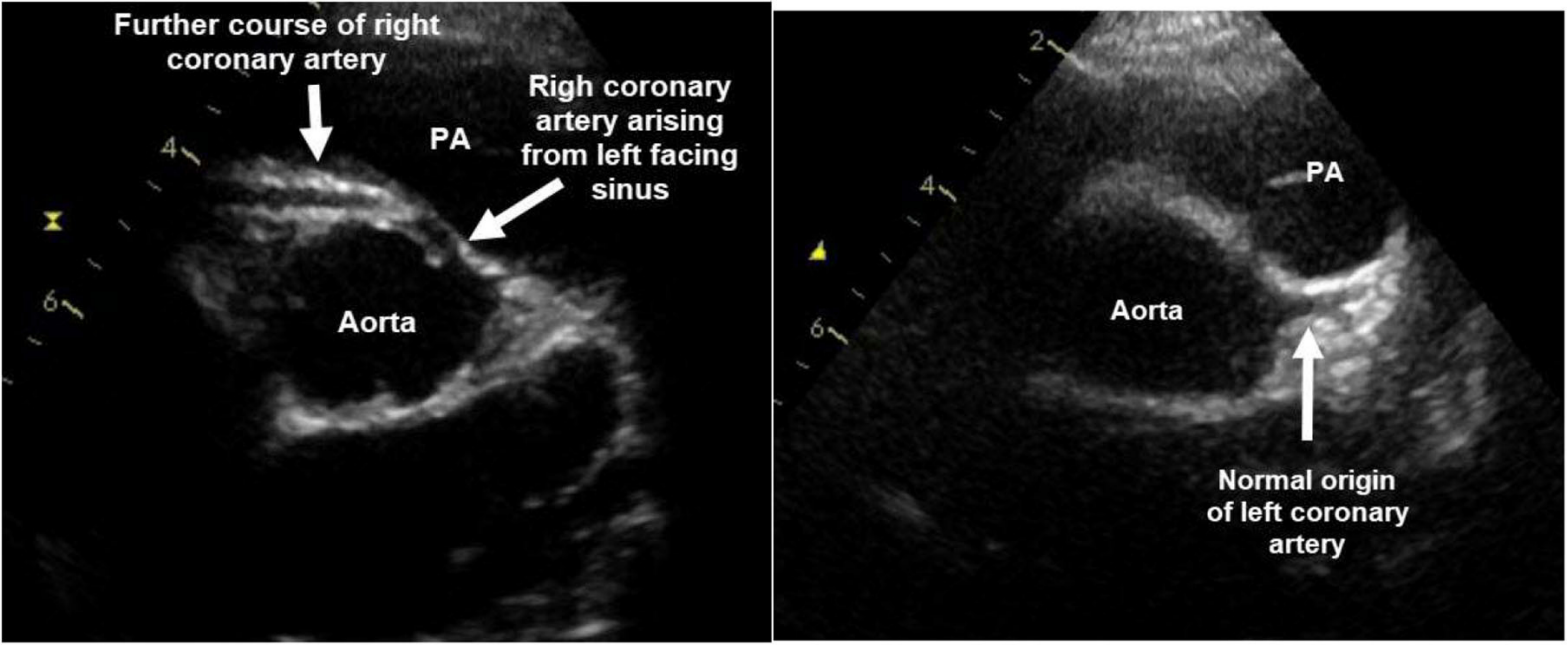
Echocardiography of patient 2: Origin of the right coronary artery (RCA) from the left-facing sinus with an interarterial and intramural course between the aorta and the pulmonary artery (PA). Normal origin of the left coronary artery from the left-facing sinus.

**Figure 5:**
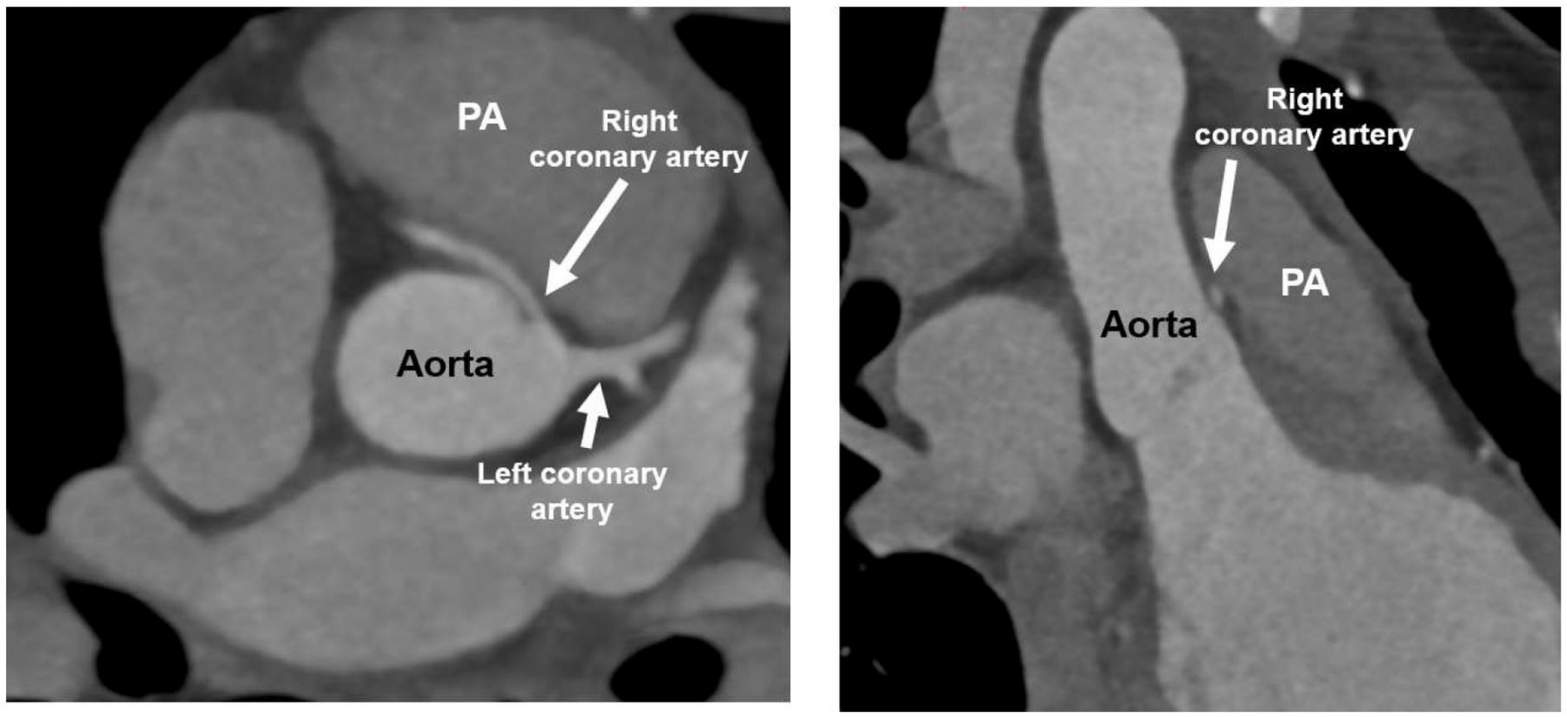
Ultra-low dose coronary computed tomography angiography (CCTA) of patient 2: AAOCA with an origin of the RCA from the left-facing sinus with an interarterial and intramural course between the aorta and the pulmonary artery and a right coronary dominance. PA, pulmonary artery.

An otherwise healthy 15-year old girl was admitted with recurring typical and atypical chest pain. She complained of left sided chest pain, especially in the evenings and at night, but also during exercise, which resulted in a reduced physical performance. Her father had died of a sudden cardiac death of unknown underlying cause. In the echocardiography, a coronary artery anomaly of the right coronary artery was suspected (Figure 4). The left- and right-ventricular dimensions and functions were normal. Resting ECG and exercise ECG showed no signs of ischemia. When using dobutamine/atropine stress Cardiac Magnetic Resonance (CMR), where a heartrate of 170 bpm was reached, the patient was symptomatic but no wall motion abnormalities were detected with the submaximal hear rate of 83% of the predicted maximum. CCTA confirmed an AAOCA with an origin of the RCA from the left-facing sinus with an interarterial and intramural course between the aorta and the pulmonary artery and a right coronary dominance (Figure 5). Because of the symptoms suggesting ischemia and the coronary artery anomaly with anatomic high-risk features, operation with unroofing of the RCA was performed. Unroofing of the RCA was executed in a similar manner to unroofing of the LCA, as explained and illustrated above. In the follow-up, 4 years later, the patient remains symptom free and shows no signs of ischemia in the ECG or echocardiography.

### Case 3

**Figure 6:**
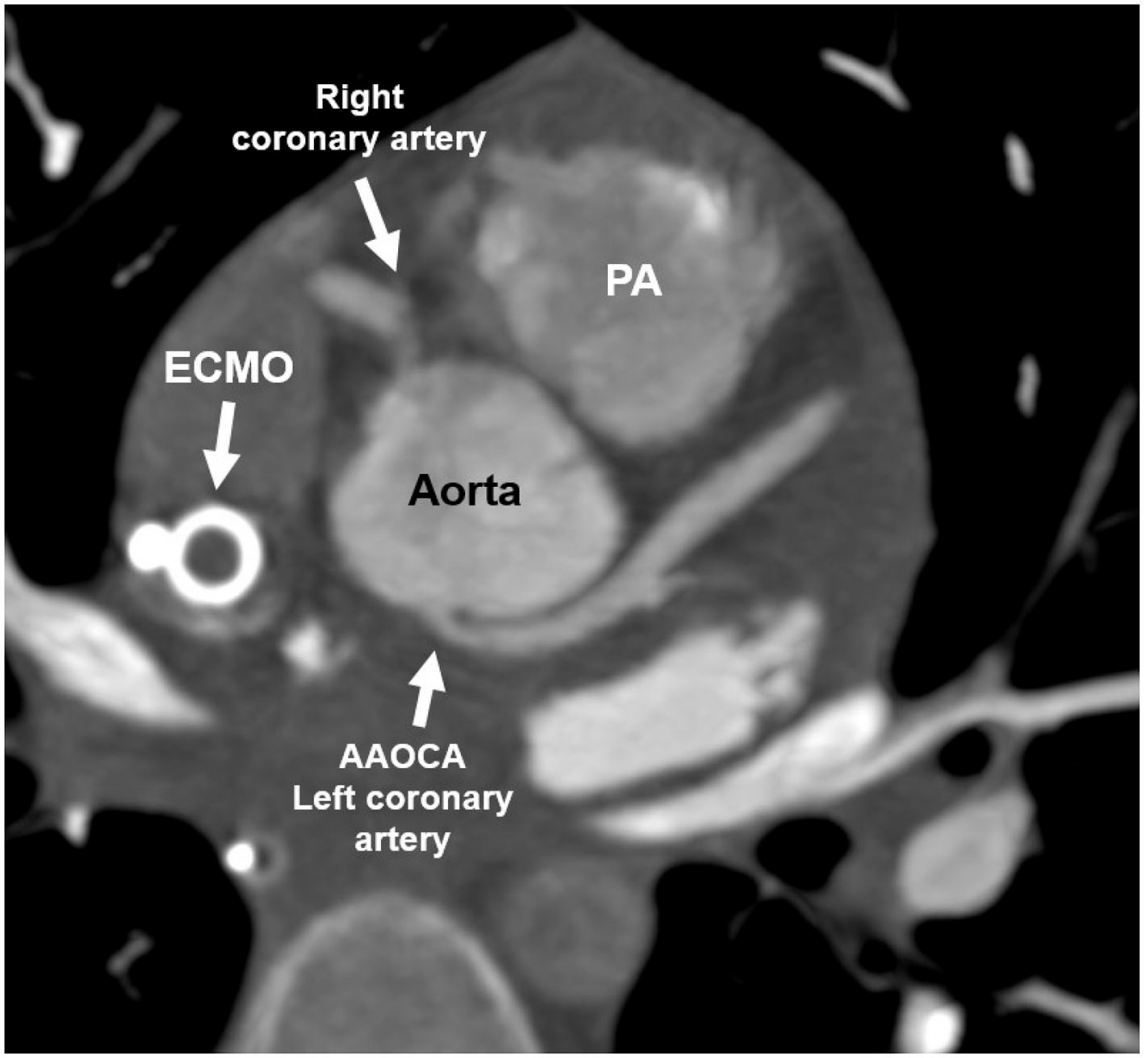
Coronary computed tomography angiography of patient 3: AAOCA with an anomalous origin of the left coronary artery arising from the non-facing sinus without an interarterial course, but with a short intramural course. ECMO, Extracorporeal membrane oxygenation; PA, pulmonary artery.

An 11-year old girl was admitted to the hospital because of a syncope after swimming. In the medical history, she reported recurring dizziness during exercise in the previous months. Laboratory results showed elevated Troponin T levels. In the initial echocardiography, left ventricular function was reduced to an ejection fraction of 33% with hypokinesia especially of the apex and posterior wall. The patient was hospitalized with suspected myocarditis. On day 4 she developed ventricular fibrillation resulting in cardiopulmonary resuscitation. After return to spontaneous circulation hemodynamic instability persisted, with the need of extracorporeal membrane oxygenation (ECMO) support. An invasive coronary angiography and an additional CCTA under ECMO showed an AAOCA with an anomalous origin of the left coronary artery arising from the non-facing sinus and the suspicion of a short intramural course of the LCA which may have resulted in coronary ischemia (Figure 6). Intraoperatively a long 1 cm segment of an intramural course was confirmed and unroofing was performed. After operation of the LCA, ECMO could be weaned off. Otherwise, the cardiac work-up was unremarkable and initial wall motion abnormalities in the territory of the left coronary artery and the subsequent ventricular fibrillation with heart failure was interpreted due to the prolonged ischemia induced by the coronary artery anomaly. The patient remained symptomatic with congestive heart failure and there was no sign of myocardial recovery over month. Therefore, a biventricular assist device was implanted and she was listed for heart transplantation, which was successfully performed 3 month later.

## Discussion

We are reporting on three cases of teenagers, all presenting with an AAOCA and an interarterial and/or intramural course. All three cases showed typical exercise induced symptoms like chest pain, syncope or dizziness. AAOCA is a rare condition with a prevalence of around 1% in the general population (4) and with different variants and relevance, depending on the subsequent course of the anomalous coronary artery. As certain subtypes (those with interarterial/intramural course of the coronary artery) are possibly associated with myocardial ischemia and sudden cardiac death, it is crucial to identify patients at risk, in order to provide optimal care prior to irreversible myocardial damage (1).

This can be difficult as many teenagers with chest pain, syncope and dizziness are seen in pediatric outpatient -and emergency departments. In this age group chest pain is mostly idiopathic or musculoskeletal (5) and also syncope and dizziness are mostly benign in etiology (6). Nevertheless, it is crucial to recognize the red flag associated with exercise induced symptoms (chest pain, dizziness, syncope) in this age group and the need for further diagnostics in these patients.

The thorough work-up, including laboratory testing's, ECG and additional imaging (i.e., echocardiography followed by CMR and/or CT) in order to assess coronary artery anatomy is crucial in this clinical setting to identify the underlying cause. Indeed, only two patients showed elevated Troponin T levels shortly after occurrence of symptoms; in addition, resting ECG and echocardiography can even be misleading, especially because resting ECG does not always show signs of ischemia. Echocardiography can show a broad spectrum from normal function to severely reduced ventricular function; the additional problem is, that coronary arteries, especially after the infant period, are often difficult to view in echocardiography. An important and more frequently seen differential diagnosis in teenagers with cardiac symptoms and elevated troponins in combination with and normal or impaired ventricular function, represents acute myocarditis. Therefore, if suspected, further imaging is imperatively needed to exclude myocarditis and to clearly depict the coronary artery anatomy and their course. CMR is the ideal tool to diagnose myocarditis and also to depict coronary artery anatomy. CCTA is, depending on the institution, the first line non-invasive imaging modality to depict in high spatial and temporal resolution the coronary artery anatomy (7). Unroofing is the procedure of choice to correct the intramural segment of AAOCA in young patients. In our case and in accordance to other reports (8), outcome after unroofing is favorable with low risk of surgery and excellent intermediate-term survival.

## Conclusion

In conclusion, even though infrequent, AAOCA in teenagers is a relevant and potentially life threatening congenital anomaly, which should be diagnosed and treated accordingly.

## Data Availability Statement

The original contributions presented in the study are included in the article/supplementary material, further inquiries can be directed to the corresponding author/s.

## Ethics Statement

Written informed consent was obtained from the individual(s), and minor(s)' legal guardian/next of kin, for the publication of any potentially identifiable images or data included in this article.

## Author Contributions

JB and J-PP drafted the initial manuscript. CG, AK, and MG critically reviewed the manuscript. All authors approved the final manuscript.

## Conflict of Interest

The authors declare that the research was conducted in the absence of any commercial or financial relationships that could be construed as a potential conflict of interest.
